# MTOR inhibition enhances NVP-AUY922-induced autophagy-mediated KIT degradation and cytotoxicity in imatinib-resistant gastrointestinal stromal tumors

**DOI:** 10.18632/oncotarget.2607

**Published:** 2014-10-21

**Authors:** Yuan-Shuo Hsueh, Hui Hua Chang, Nai-Jung Chiang, Chueh-Chuan Yen, Chien-Feng Li, Li-Tzong Chen

**Affiliations:** ^1^ National Institute of Cancer Research, National Health Research Institutes, Tainan, Taiwan; ^2^ Institute of Clinical Pharmacy and Pharmaceutical Sciences, College of Medicine, National Cheng Kung University, Tainan, Taiwan; ^3^ Department of Internal Medicine, National Cheng Kung University Hospital, Tainan, Taiwan; ^4^ Division of Hematology and Oncology, Department of Medicine, Taipei Veterans General Hospital, Taipei, Taiwan; ^5^ National Yang-Ming University School of Medicine, Taipei, Taiwan; ^6^ Department of Pathology, Chi-Mei Foundation Medical Center, Tainan, Taiwan; ^7^ Department of Biotechnology, Southern Taiwan University of Science and Technology, Tainan, Taiwan; ^8^ Graduate Institute of Medicine, College of Medicine, Kaohsiung Medical University, Kaohsiung, Taiwan; ^9^ Department of Internal Medicine, Kaohsiung Medical University Hospital, Kaohsiung Medical University, Kaohsiung, Taiwann

**Keywords:** gastrointestinal stromal tumor, KIT, heat shock protein 90 inhibitor, MTOR inhibitor, autophagy

## Abstract

Our previous study demonstrated NVP-AUY922, a HSP90AA1 inhibitor, could enhance mutant KIT degradation in gastrointestinal stromal tumor (GIST) cells through both proteasome- and autophagy-mediated pathways. Herein, we showed rapamycin, a MTOR inhibitor and autophagy inducer, could reduce total and phospho-KIT expression levels and enhance apoptosis in imatinib-resistant GIST cells. The involvement of autophagy in rapamycin-induced KIT downregulation was further confirmed by co-localization of KIT and autophagosome, and partial recovery of KIT expression level by either siRNA-mediated BECN1 and ATG5 silencing or autophagy inhibitors after rapamycin. Rapamycin and NVP-AUY922 synergistically inhibited GIST cells growth *in vitro*. The combination of low-dose NVP-AUY922 with rapamycin had comparable effects on reducing KIT expression, increasing MAP1LC3B puncta and tumor necrosis, and inhibiting tumor growth as high-dose NVP-AUY922 did in GIST430 xenograft model. Our results suggest the addition of a MTOR inhibitor may reduce NVP-AUY922 dose requirement and potentially improve its therapeutic index in mutant KIT-expressing GISTs.

## INTRODUCTION

Gastrointestinal stromal tumors (GISTs) are the most common type of mesenchymal neoplasms in the gastrointestinal tract [1, 2]. Approximately 85% of GISTs harbor gain-of-function mutations in *KIT* or *PDGFRA*, which lead to the promotion of cell survival and escape from apoptosis. In GISTs, *KIT* mutations occur mainly in the exon 11 juxtamembrane domain, followed by the exon 9 extracellular domain, the exon 13/14 ATP-binding domain, and the exon 17 activation loop domain [3-5]. Imatinib mesylate (IM; Gleevec®, Novartis Pharma, Basel, Switzerland) and sunitinib malate (SU; Sutent®, Pfizer Inc., CA, USA) are the first-line and second-line drugs for metastatic/unresectable GISTs and IM-resistant GISTs, respectively [6, 7]. In a pivotal phase III trial, regorafenib yielded notable improvement in progression-free survival in IM/SU failure patients comparing to placebo control and has recently been approved as a third-line drug for IM/SU-resistant GISTs [8]. Unfortunately, TKI resistance remains an increasing issue after long-term tyrosine kinase inhibitor (TKI) treatment. HSP90AA1, a chaperone protein that assists the folding and maturation of its client proteins, is an alternative therapeutic target for cancer therapy [9-11]. Inhibition of HSP90AA1 by 17-AAG, the first HSP90AA1 inhibitor tested in clinical trials, led to KIT downregulation and cell death in both mutant KIT-expressing mast cells and GIST cell lines [12, 13].

However, 17-AAG has several pharmacological limitations, including poor bioavailability, difficulty in formulation, and hepatotoxicity to prevent its further application in clinical setting. Therefore, we evaluated the anti-proliferation effects of a next-generation HSP90AA1 inhibitor, NVP-AUY922 (AUY922), which has high affinity against HSP90AA1 *in vitro,* for mutant KIT expressing GIST cell in our previous study [14-17]. In that study, AUY922 effectively downregulated both total and phosphorylated KIT and induced cell apoptosis in both IM-sensitive and IM-resistant GIST cells. However, it was surprisingly to find that AUY922-induced KIT reduction as well as endogenous KIT turnover, were mediated by both autophagy and proteasome degradation pathways. These results highlight the feasibility of AUY922 in the treatment of mutant KIT-expressing GISTs and the novel role of autophagy in endogenous and AUY922-induced KIT degradation. However, despite the high antitumor activity of AUY922 against GIST cells, AUY922 therapy at dose of 70 mg/m^2^ weekly infusion, the maximum tolerated dose defined in phase I trial, was associated with unneglectable ocular adverse events, including night blindness, photopsia, blurred vision and visual impairment [18].

Based on our previous findings, we hypothesize that the combination of AUY922 with an autophagy inducer that may synergistically or additively enhance KIT downregulation, and thus diminish the dose of AUY922 for GIST treatment and subsequently minimize the incidence and severity of ocular adverse events. Classically, mammalian target of rapamycin (MTOR) kinase is the well-known modulator of autophagy in human cells. Inhibition of MTOR leading to autophagy activation has been demonstrated as a therapeutic mechanism for various cancer types [19-22]. Rapamycin, a MTOR inhibitor that widely used as an immunosuppressant in organ-transplanted patients, was able to induce autophagy and enhance degradation of aggregate-prone proteins, including huntingtin in several Huntington's disease models [23-25]. Moreover, rapamycin has also been demonstrated antitumor activity through the induction of autophagy in malignant gliomas and chronic myeloid leukemia [21, 22]. Numerous clinical trials are undergoing to investigating its effects as autophagy modulators either alone or in combination with standard drug therapy for various cancer types, including pancreatic cancer, advanced solid tumor, multiple myeloma, and melanoma [26].

In this study, we investigated whether the combination of AUY922 and rapamycin would be a potential strategy to improve the therapeutic index of AUT922 in mutant KIT-expressing GISTs. We evaluated the effect of rapamycin alone and the potential synergism between AUY922 and rapamycin on induction of autophagy activation, KIT reduction and growth inhibition in IM-resistant, mutant KIT-expression GIST cells both *in vitro* and *in vivo*. These results represent a strategy toward optimizing the use of AUY922 for TKI-resistant GISTs.

## RESULTS

### Rapamycin induced autophagy, downregulated KIT expression, and led to cell apoptosis

**Figure 1:**
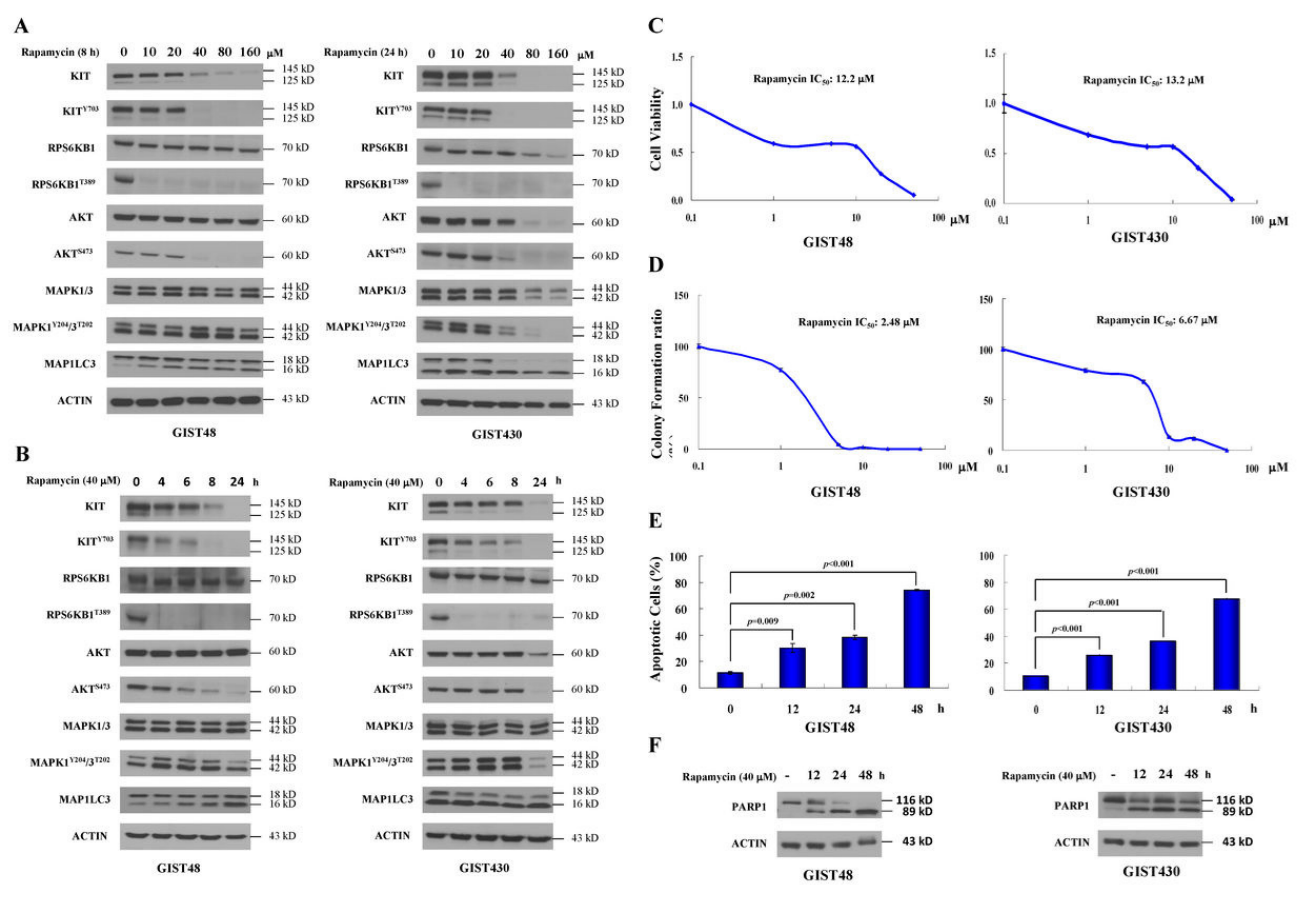
Rapamycin showed antitumor activity and reduced KIT expression in GIST430 and GIST48 cells GIST430 and GIST48 cells were treated with rapamycin at the indicated doses (A) and times (B) and analyzed by immunoblotting against phospho- and total KIT, RPS6KB1, MAPK1/3, AKT, or MAP1LC3 proteins. GIST430 and GIST48 cells were incubated with rapamycin at the indicated doses, and IC_50_ was determined by the cell viability assay (C) and clonogenic assay (D). GIST430 and GIST48 cells were treated with 40 μM rapamycin from 12 to 48 h and then analyzed by Annexin V staining (E) or immunoblotting against PARP1 (F). All experiments were repeated at least three times. The data are expressed as the mean ± S.E. of two or more independent experiments.

Initially, we examined the inhibitory effects of rapamycin in GIST cells. The data showed that 10 μM rapamycin effectively reduced phospho-RPS6KB1, a downstream MTOR target molecule, and that 10 to 40 μM rapamycin induced clear MAP1LC3B accumulation, an index of autophagy activity, in both GIST48 and GIST430 cells. (Fig. 1A) These findings were accompanied by downregulation of phospho- and total KIT and KIT-modulated phospho-AKT in both GIST48 and GIST430 cells, but phospho-MAPK1/3 only in GIST430 cells. In the time series experiments, 40 μM rapamycin abolished phospho-RPS6KB1 level after 4 h of exposure in both GIST cell lines. (Fig. 1B) Phospho-KIT and phospho-AKT were clearly downregulated at 8 h in GIST48 cells and at 24 h in GIST430 cells, which was consistent with the time course of MAP1LC3B accumulation. In GIST430 cells, 40 μM rapamycin reduced phospho-MAPK1/3 at 24 h, which was consistent with phospho-KIT downregulation, but phospho-MAPK1/3 was not decreased after rapamycin treatment in GIST48 cells. These results imply that phospho-MAPK1/3 could be activated by other signaling cascades in GIST48 cells.

The rapamycin drug concentrations that inhibited cell viability by 50% (IC_50_) were 12.2 and 13.2 μM for GIST48 and GIST430 cells, respectively. (Fig. 1C) In the colony formation assay, the IC_50_ of rapamycin were 2.48 μM and 6.67 μM for GIST48 and GIST430 cells, respectively. (Fig. 1D) Then, we examined the effect of rapamycin on the induction of GIST cell death. Fig. 1E showed that in GIST48 cells, 40 μM rapamycin induced Annexin V-positive cells in 11.50%, 30.10%, 38.52%, and 74.09% of total cells after 0, 12, 24, and 48 h of incubation, respectively, and Annexin V-positive/PI-negative cells were 4.72%, 17.08%, 6.24%, and 5.52%, respectively. For GIST430 cells, the Annexin V-positive cells constituted 10.37%, 26.06%, 36.07%, and 67.74% of total cells, respectively, and Annexin V-positive/PI-negative cells constituted 3.86%, 11.98%, 15.19%, and 24.79% of cells after 0, 12, 24, and 48 h of 40 μM rapamycin incubation, respectively. Moreover, rapamycin induced PARP1 cleavage in the same treatment condition in both GIST cell lines. (Fig. 1F) These results demonstrate that rapamycin alone could downregulate phospho- and total KIT and induce apoptosis.

### Rapamycin-induced KIT reduction occurred through enhancement of autophagy

**Figure 2:**
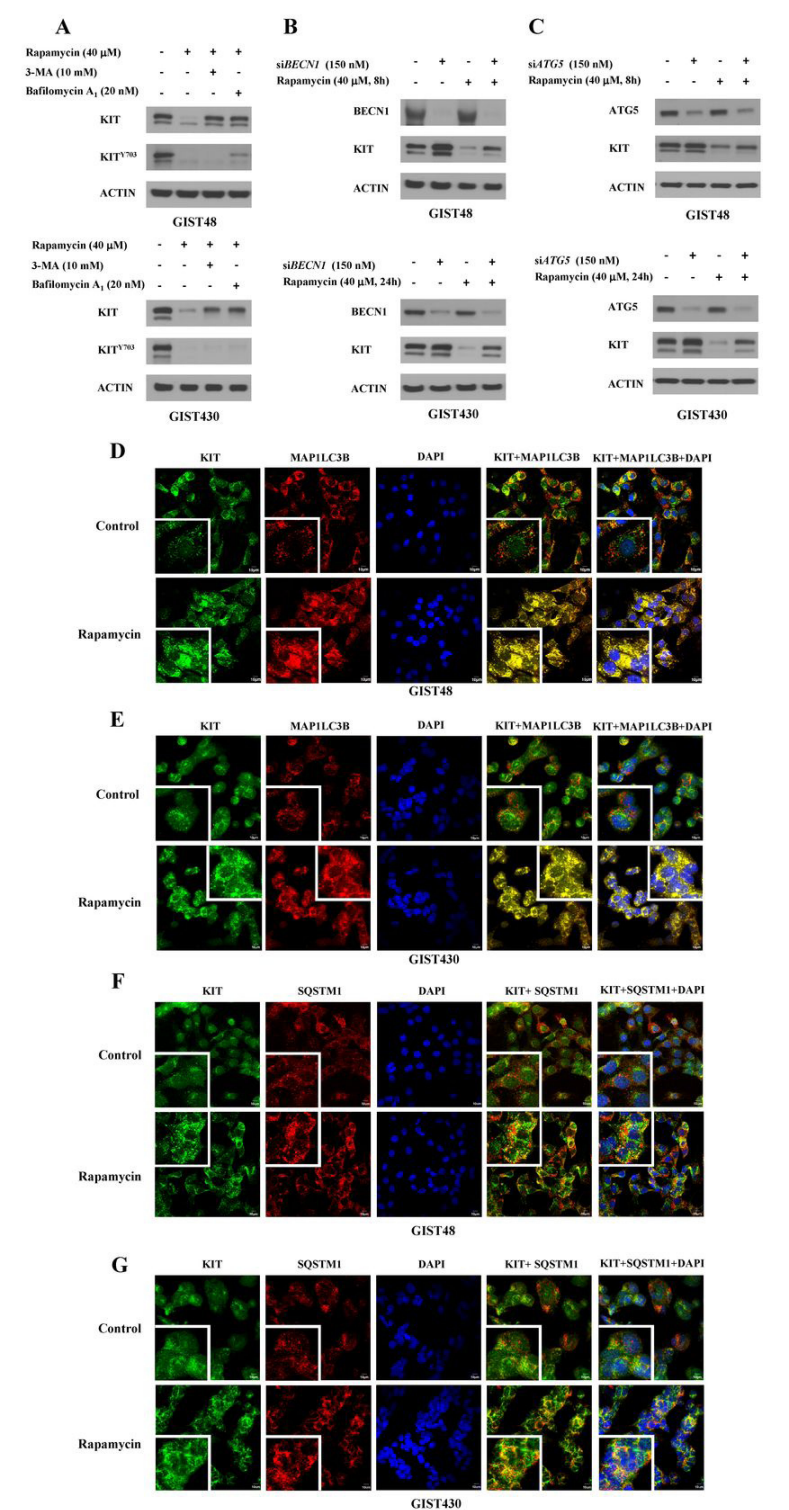
Rapamycin-induced KIT downregulation via autophagy activation GIST430 and GIST48 cells were pretreated with 10 mM 3-MA and 20 nM bafilomycin A_1_ (A) for 4 h and then treated with 40 μM rapamycin for an additional 24 and 8 h, respectively. The cells were lysed and analyzed by immunoblotting against phospho- and total KIT. GIST430 and GIST48 cells were transfected with 150 nM siRNA targeting *BECN1* (B) or *ATG5* (C) for 72 h and then treated with 40 μM rapamycin for another 24 and 8 h, respectively. Cell lysates were extracted and analyzed by immunoblotting against BECN1, ATG5, and KIT. GIST430 and GIST48 cells were treated with 40 μM rapamycin for 24 and 4 h, respectively, and then stained with KIT, MAP1LC3B (D, E), or SQSTM1 (F, G). After immunostaining, cells were visualized by confocal microscopy, and images were acquired through the Cy2, Rhodamine, or DAPI channels (600 x). The inserted figure in the corner showed magnified (2400 x) and representative cells of each image. The data were representative images of 5 fields/pictures for each sample.

Because the inhibition of MTOR influenced protein synthesis, transcription, and autophagy activity, we investigated whether the rapamycin-induced KIT reduction is a result of autophagy activation. Cells were pretreated with the autophagy inhibitor 3-MA or bafilomycin A_1_ for 4 h and then incubated with 40 μM rapamycin for an additional 8 and 24 h for GIST48 and GIST430 cells, respectively. The data showed that rapamycin-induced KIT downregulation could be partially rescued by 3-MA or bafilomycin A_1_ in both GIST48 and GIST430 cells. (Fig. 2A) To further confirm the role of autophagy in rapamycin-induced KIT downregulation, we independently silenced the expression of two proteins essential for autophagosome formation, BECN1 and ATG5, and found that rapamycin-induced KIT reduction was diminished in BECN1- or ATG5-deficient GIST48 and GIST430 cells. (Fig. 2B and C) Using immunofluorescence staining and confocal microscopy analysis, we found no obvious nonspecific staining in normal rabbit, goat, or mouse antibody-incubated cells. After treatment of both GIST cells, rapamycin clearly led to more puncta of MAP1LC3B and more aggregates of SQSTM1, an index of autophagosome, as well as KIT condensation and aggregation. (Fig. 2D-G) Furthermore, the aggregates of SQSTM1 and accumulation of MAP1LC3B-labeled autophagosomes co-localized with KIT in the autophagosomes that were visualized as yellow foci. These results indicate that rapamycin enhanced autophagy activity and led to KIT downregulation.

### AUY922 downregulated phospho- and total KIT expression and induced apoptosis

**Figure 3:**
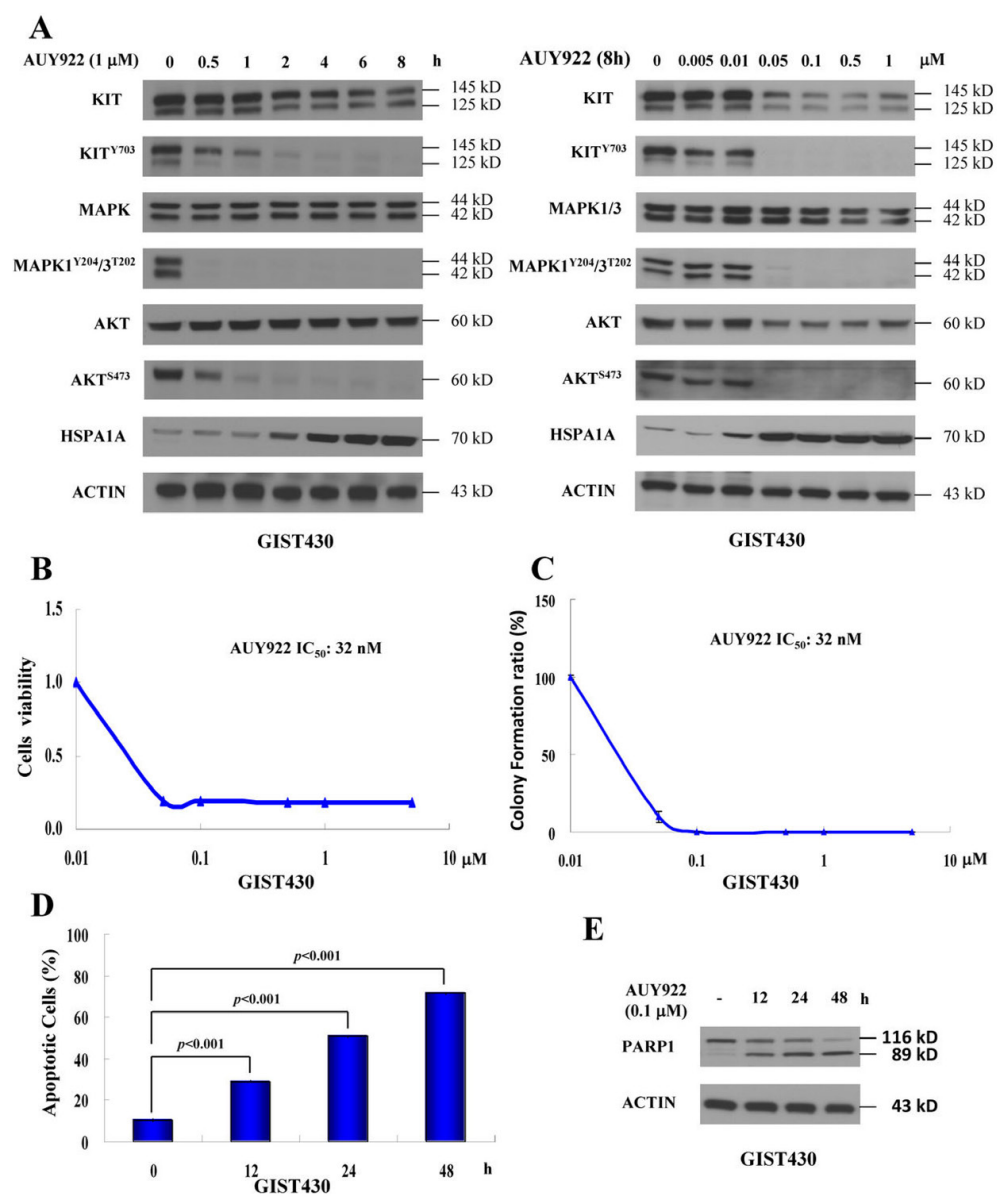
AUY922 reduced KIT expression and induced apoptosis in GIST430 cells GIST430 cells were treated with AUY922 at the indicated doses and times (A) and analyzed by immunoblotting against phospho- and total KIT, MAPK1/3, AKT, or HSPA1A proteins. GIST430 cells were incubated with AUY922 at the indicated doses and IC_50_ was determined by the cell viability assay (B) or clonogenic assay (C). Cells were treated with 0.1 μM AUY922 for 12 to 48 h and then analyzed by Annexin V staining (D) or immunoblotting against PARP1 (E). All experiments were repeated at least three times. The data are expressed as the mean ± S.E. of two or more independent experiments.

In previous studies, we demonstrated that AUY922 reduces KIT expression in IM-sensitive GIST882 and IM-resistant GIST48 cells. In the present study, we first assessed whether AUY922 could reduce phospho- and total KIT in another IM-resistant GIST430 cells. The data showed that 1 μM AUY922 elevated the level of HSPA1A expression in a time-dependent manner, which is a positive indicator of HSP90AA1 inhibition. (Fig. 3A) This result suggests that AUY922 effectively inhibited HSP90AA1 activity in GIST430 cells. Moreover, AUY922 downregulated the phospho- and total KIT expression levels in GIST430 cells after incubation for 0.5 h. KIT downstream signaling was assessed by levels of phospho-MAPK and phospho-AKT and was found to be inactivated, consistent with phospho-KIT suppression. After drug exposure for 8 h, AUY922 elevated HSPA1A expression in a dose-dependent manner in a range from 0.05 to 1 μM, and it simultaneously reduced phospho- and total KIT proteins in GIST430 cells. (Fig. 3A) Consistent with phospho-KIT downregulation, the phospho-MAPK1/3 and phospho-AKT levels were also decreased in GIST430 cells.

To examine the antitumor activity of AUY922 in GIST430 cells, cells were incubated with AUY922 and then analyzed using the cell viability assay (Fig. 3B) and the clonogenic assay. (Fig. 3C) The IC_50_ of AUY922 was 32 nM for both the cell viability assay and clonogenic assay. Then, we treated GIST430 cells with 0.1 μM AUY922 for 12 to 48 h to determine whether AUY922 could induce apoptosis. After AUY922 treatment for 0, 12, 24, or 48 h, Annexin V-positive cells were in 10.52%, 29.05%, 50.64%, and 71.54% of total cell population, respectively, and Annexin V-positive/PI-negative cells were in 5.63%, 7.58%, 16.88%, and 26.63% of total cell population, respectively. (Fig. 3D) Furthermore, 0.1 μM AUY922 induced PARP1 cleavage with incubation times ranging from 12 to 48 h. (Fig. 3E) These findings indicate that AUY922 is highly effective in reducing KIT expression in GIST430 cells, similar to its previously reported effects in GIST48 and GIST882 cells.

### Rapamycin enhanced AUY922-induced KIT downregulation and cell death

**Figure 4:**
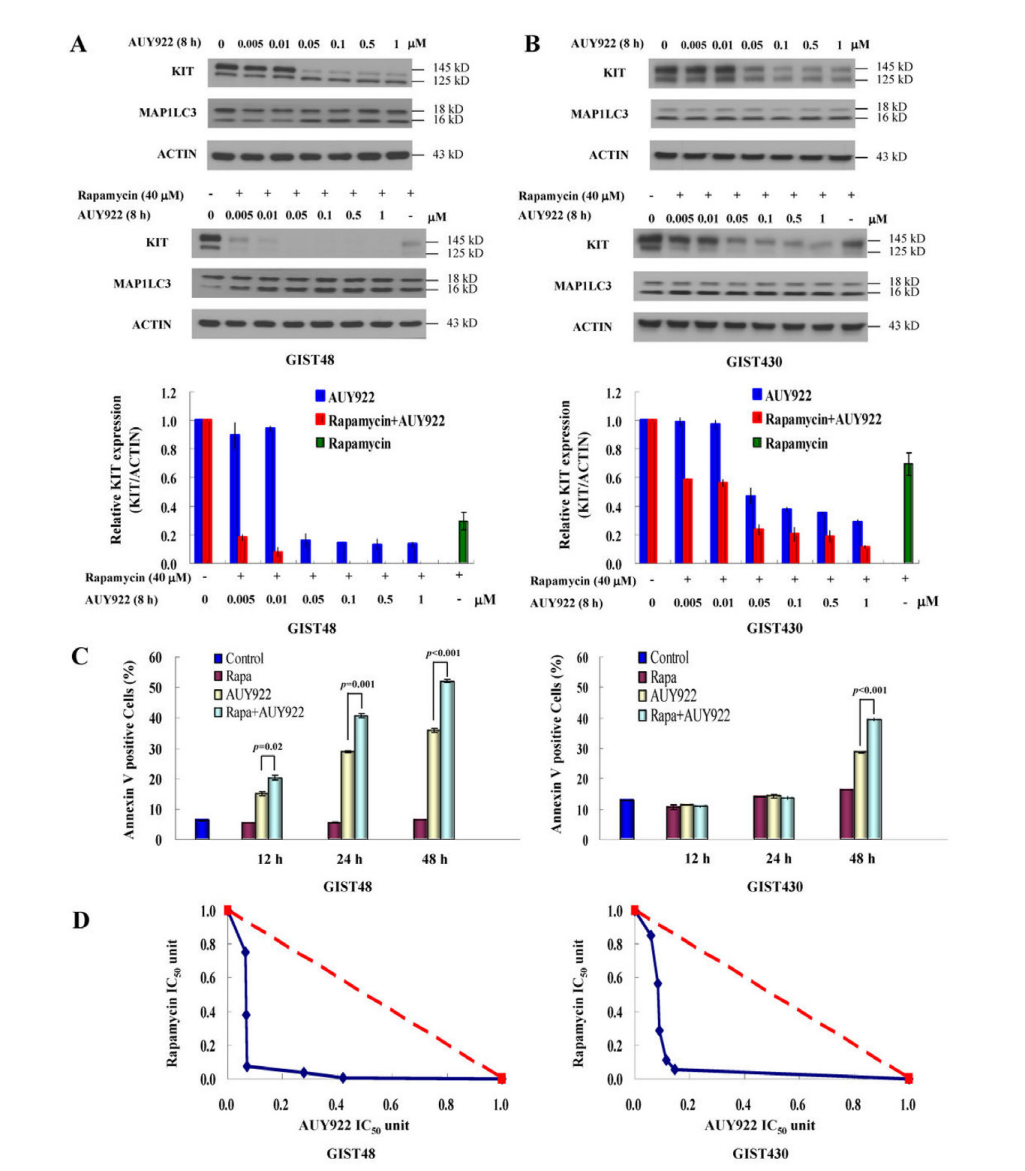
The combination effect of AUY922 and rapamycin on KIT protein degradation in GIST430 and GIST48 cells GIST430 (A) and GIST48 (B) cells were treated with the indicated dose of AUY922 alone or in a combination with 40 μM rapamycin for 8 h. Cells were lysed and analyzed by immunoblotting against KIT and MAP1LC3. Relative KIT expression was normalized to ACTIN and compared with that of the untreated control. Cells were treated with 20 nM AUY922 and 5 μM rapamycin, either alone or in combination, for 12 to 48 h and analyzed by Annexin V staining (C). Cells were treated with various concentrations of AUY922 combined with rapamycin (D). Cell growth was determined by the methylene blue dye assay as described in the Materials and Methods section. The interaction between AUY922 and rapamycin at the IC_50_ point was analyzed using the isobologram method. The data are expressed as the mean ± S.E. of two or more independent experiments.

Rapamycin alone showed its ability to induce KIT reduction and cell death via autophagy activation. Next, we examined whether the drug combination of rapamycin and AUY922 could achieve better effects on KIT downregulation and cell death than each drug treatment alone. In GIST48 cells, 0.005 to 1 μM AUY922 combined with 40 μM rapamycin more robustly reduced KIT expression after 8 h of incubation than rapamycin or AUY922 alone. (Fig. 4A) In GIST430 cells, the drug combination of 0.005 to 1 μM AUY922/40 μM rapamycin similarly had stronger effects on KIT downregulation than each drug independently. (Fig. 4B) In GIST48 cells, 20 nM AUY922/5 μM rapamycin treatment increased the proportion of Annexin V-positive cells from 6.27% to 20.33%, 40.78%, and 52.13% after 12, 24, and 48 h of incubation, respectively, as compared with that of after either rapamycin (5.38%, 5.54%, and 6.36%) or AUY922 (15.04%, 28.75%, and 35.93%) alone. (Fig. 4C) In GIST430 cells, the Annexin V-positive cell fraction did not significantly altered after 12 or 24 h exposure to study drugs, but it increased to 39.56% after 48 h incubation with 20 nM AUY922/5 μM rapamycin as compared with that of 16.18% and 28.7% after 5 μM rapamycin alone and 20 nM AUY922 alone, respectively. To further evaluate the potential synergy between AUY922 and rapamycin, the effect of drug combination on cell viability in GIST48 and GIST430 cells was analyzed. Fig. 4D showed that the drug combination achieved a lower IC_50_ than either drug alone, implying that AUY922 and rapamycin had a synergistic effect on the viability of both GIST48 and GIST430 cells. In summary, rapamycin enhances AUY922-induced KIT downregulation, cell apoptosis, and cell viability in GIST cells.

### The combination of low-dose AUY922 and rapamycin achieved comparable tumor growth inhibition as high-dose AUY922

**Figure 5:**
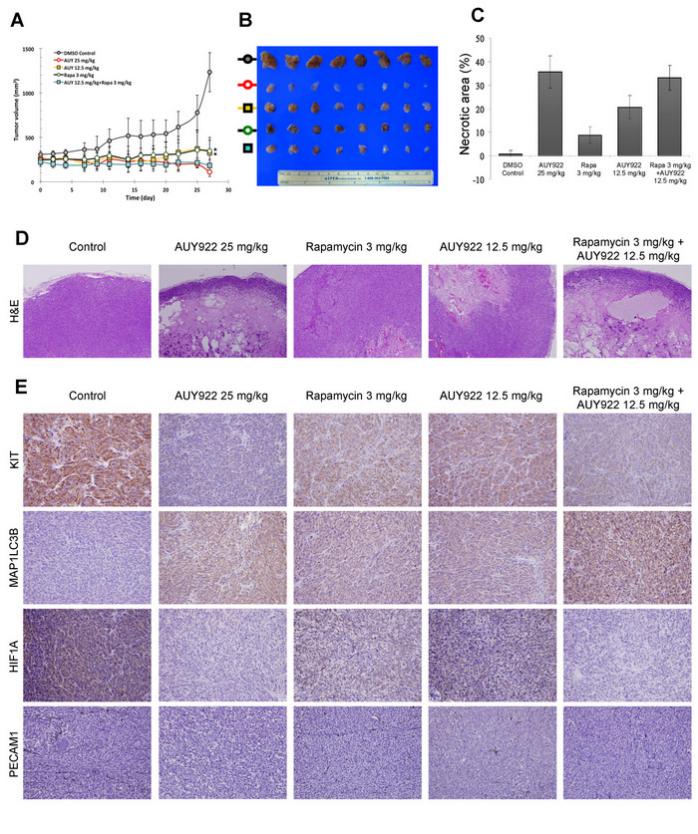
The effect of AUY922 either alone or in a combination with rapamycin in the GIST430 xenograft animal model The GIST430 xenograft was established as described in Materials and Methods. Mice received an intraperitoneal injection of DMSO, AUY922 25 mg/kg twice weekly, AUY922 12.5 mg/kg twice weekly, rapamycin 3 mg/kg twice weekly, or a combination of AUY922 and rapamycin for 4 weeks. (A) The tumor volume was determined as 1/2 x length (mm) x width (mm)^2^. The dissected tumors were collected at the end of drug administration (B) and then analyzed by H&E staining (D) and immunohistochemical staining against KIT, MAP1LC3B, HIF1A, or PECAM1 (E). The necrotic areas of the tumor specimens were determined by quantification, as shown in (C). The data are expressed as the mean ± S.D. of two or more independent experiments. * *p* value is less than 0.05.

The synergism between AUY922 and rapamycin in inhibiting the growth of GIST cells *in vitro* was further evaluated in GIST430 xenograft animal model. Based on previous studies of AUY922 for cancer xenograft models, AUY922 at a dose of 25 mg/kg (high-dose) twice weekly *i.p.* for 4 weeks was firstly explored. Our results showed that mice with 25 mg/kg b.i.w. of AUY922 treatment had significantly lower tumor volumes as compared with the DMSO-treated control group (data not shown). To assess drug synergism, the concentration of AUY922 was reduced to 12.5 mg/kg (low-dose) in a combination with rapamycin for experimental arms. Mice were treated with DMSO (placebo control), high-dose AUY922 b.i.w. (active control), and low-dose AUY922 b.i.w., 3 mg/kg rapamycin b.i.w., or a combination of both. The mean tumor volumes in corresponding group of mice were 1232.94, 112.00, 325.71, 348.31, and 185.03 mm^3^, respectively. (Fig. 5A) At the end of treatment, the tumor volume of mice with high-dose AUY922 was significantly smaller than those with low-dose AUY922 (*p*= 0.004) or rapamycin (*p*= 0.002). While the mean tumor volume was comparable, there is no significantly different between mice treated with high-dose AUY922 and those with low-dose AUY922 and rapamycin combo. Immunohistochemical staining of tumor tissue sections from the GIST430 xenograft revealed that low-dose AUY922/rapamycin combo, as well as high-dose AUY922, induced more prominent tumor necrosis, as visualized by hematoxylin and eosin (H&E) staining, and achieved a more pronounced effect on KIT downregulation and MAP1LC3B accumulation than low-dose AUY922 or rapamycin alone *in vivo*. (Fig. 5C-E) Moreover, a marker of angiogenesis (platelet/endothelial cell adhesion molecule 1, PECAM1) and a key component of this pathway (hypoxia inducible factor 1A, HIF1A) were both more obviously reduced in the tumor tissue from the high-dose AUY922 alone and low-dose AUY922/rapamycin treated mice compared with those from each treatment alone.

## DISCUSSION

Recently, HSP90AA1 has been shown to be a novel target for cancer therapy because its inhibition can enhance the degradation of its client proteins, typically receptor tyrosine kinases, signaling molecules and kinases, and transcription factors, to result in modulating many crucial cell functions, including survival, proliferation, metastasis, angiogenesis, cell cycle regulation, and apoptosis [27]. Although many HSP90AA1 inhibitors exhibited antitumor activity against GIST *in vitro*, only IPI-504, STA-9090, BIIB021, and AUY922 have been evaluated as single-agent therapies for GIST refractory to IM and SU *in vivo* [28-31]. IPI-504, a derivative of 17-AAG, has displayed antitumor activity against TKI-refractory GISTs, but its clinical trial was early terminated because of the occurrence of on-treatment deaths along with severe unexpected adverse events including renal and liver failure, metabolic acidosis, and cardiopulmonary arrest [28, 32]. Of the phase II study of STA-9090 in patients with metastatic GISTs showed that weekly administration of STA-9090 was not sufficient to inhibit KIT activity and its downstream pathways [33]. BIIB021 achieved a 22% overall metabolic response rate according to 18 fluoro-2-deoxyglucose positron emission tomography (FDG-PET), while the objective tumor response rate was 0% and 4% by RECIST and Choi's criteria, respectively [34]. AUY922, one of the most potent HSP90AA1 inhibitors, is currently under clinical investigation for TKI-refractory GIST. However, the occurrence of ocular adverse events, notably night blindness, frequently leads to treatment interruption and/or dose reduction in our phase II study. Developing a novel strategy to increase the therapeutic index of HSP90AA1 inhibitor will be critical for future development of this class of compounds in patients with TKI-refractory GISTs [17]. In our previous study, we showed that AUY922-induced KIT downregulation was via both proteasome and autophagy degradation pathways. In current study, we demonstrated that rapamycin, a MTOR inhibitor and inducer of autophagy, could reduce the expression of both phospho- and total KIT in time- and dose-dependent manners, and thus enhance the apoptosis of KIT-expressing GIST cells. AUY922 and rapamycin synergistically enhanced KIT downregulation and growth inhibition as compared with individual drug alone. These findings suggest that combining AUY922 with an autophagy inducer can be a potential therapeutic strategy for TKI-refractory GISTs.

Inhibition of MTOR that leads to autophagy activation has been demonstrated as a therapeutic approach for cancers in many studies. Temsirolimus, an inhibitor of MTOR, induces autophagy and cell cycle arrest to result in proliferation inhibition of mantle cell lymphoma [19]. Another MTOR inhibitor, everolimus, also induces autophagy and cell cycle arrest and improves the median survival of mice harboring human childhood B-cell progenitor acute lymphoblastic leukemia [20]. Takeuchi *et al.* and Carayol *et al.* also showed that rapamycin exhibits an antitumor effect by inducing autophagy in malignant glioma cells and malignant chronic myeloid leukemia, respectively [21, 22]. In our study, high concentrations of rapamycin, up to 10 to 40 μM, were required to induce clear MAP1LC3B accumulation. (Fig. 1A) Typically, rapamycin is known to inhibit phospho-RPS6KB1 and cell proliferation under nM concentration [35, 36]. However, recent studies suggested that low-does (nM) and high-dose (μM) of rapamycin had different target and effects on cancer cells. Low-dose rapamycin can inhibit MTOR complex 1 (MTORC1) and lead to cell growth inhibition. On the other hand, much higher dose (at μM range) is required to suppress MTOR complex 2 (MTORC2) and induce cell apoptosis [37]. Yellen P. *et al.* showed that high-dose (μM) rapamycin-induced cell apoptosis was closely associated with the suppression of EIF4EBP1 phosphorylation [38]. Based on these findings, rapamycin induced autophagy and cell apoptosis at high-dose is probably through inhibiting MTORC2 and its downstream EIF4EBP1.

MTOR also plays a dominant role in nutrient sensing and the regulation of cell growth. Further studies have demonstrated that protein synthesis factors that lie downstream of MTOR affect cell proliferation and survival, cell-cycle progression, angiogenesis, energy metabolism, and metastasis [39]. Therefore, MTOR as a developing therapeutic target for cancer treatment with many rapamycin analogues (rapalogs) have been submitted to clinical trials in recently years. In 2007, temsirolimus was the first MTOR inhibitor to be approved by the FDA for the treatment of advanced-stage renal cell carcinoma. Subsequently, several MTOR inhibitors have been evaluated for the treatment of various cancers in phase III clinical trials, including Non-Hodgkin lymphoma, neuroendocrine tumors, gastric cancer, sarcoma, hepatocellular carcinoma, breast cancer, and etc. [40]. Everolimus, an oral form of MTOR inhibitor, has been shown to inhibit the proliferation of GIST cell in human GISTs xenograft model [41, 42]. In a phase I–II study, the combination of everolimus with IM, at dose of 2.5 mg and 600 mg daily, respectively, could achieve a median progression-free survival and overall survival of 3.5 months and 10.7 months, respectively, in patients with IM/SU-resistant GISTs [43].

In our study, we also found that combination of AUY922 and rapamycin could more effectively downregulate KIT expression and inhibit tumor growth in IM-resistant GIST cells *in vitro* and *in vivo* compared with individual drug alone. (Fig. 4 and 5. However, induction of autophagy activation and KIT downregulation may not be the only synergistic/additive effects exerted by the combination of AUY922 and rapamycin on GIST430 xenograft model in mice. As shown in Fig. 5, GIST 430 xenograft tumors in mice with either AUY922 or rapamycin alone, or in a combination had less PRCAM1 staining-positive blood vessels as well as HIF1A expression than the control tumors. HIF1A, the most potent pro-angiogenic protein that drives the expression of several angiogenic factors, including vascular endothelial growth factor (VEGF), is one of the client proteins of HSP90AA1. Inhibition of HSP90AA1 could enhance proteasome-mediated degradation of HIF1A followed by downregulation of HIF-mediated VEGF expression and angiogenesis [44, 45]. Furthermore, several studies have demonstrated that HSP90AA1 inhibitors decrease VEGF secretion from cancer cells, impair endothelial cell tubule formation *in vitro*, and reduce tumor size and vascularization *in vivo* [46, 47]. MTOR has also been shown to regulate the transcription and translation of HIF1A [48]. Inhibition of MTOR-induced anti-angiogenic and antitumor effects has been shown to mediate through the reduction of VEGF production and diminished vessel sprouting in tumors [49]. Moreover, Mercier *et al.* demonstrated that rapamycin treatment could decrease stromal content in tumor animal model [50]. Taken together, the combination of a HSP90AA1 inhibitor and a MTOR inhibitor not only activates autophagy, leading to KIT downregulation, but also likely exerts anti-angiogenic effects that to contribute to tumor growth inhibition *in vivo* in our study.

In conclusion, we have demonstrated that rapamycin can alone enhance autophagy and induce KIT protein downregulation and apoptosis in both IM-resistant GIST48 and GIST430 cells. Rapamycin-induced KIT degradation via the autophagy pathway was supported by co-localization of KIT with MAP1LC3B or SQSTM1 after drug incubation. In addition, blockage of autophagy by pharmacological inhibition or silencing of essential autophagy-related proteins could partially rescue rapamycin-induced KIT downregulation. The combination of AUY922 and rapamycin enhanced KIT reduction and GIST cell killing compared with each treatment alone. Furthermore, high-dose (25 mg/kg) of AUY922 could achieve significant better tumor growth inhibition than either low-dose (12.5 mg/kg) AUY922 or rapamycin 3 mg/kg monotherapy; while the low-dose AUY922/rapamycin combo had similar tumor growth inhibition as high-dose (25 mg/kg) of AUY922 did, as shown in Fig. 5. Our data highlight the combination of AUY922 and an autophagy inducer, such as rapamycin, is a potential treatment strategy for TKI-refractory, KIT-expressing GISTs and deserves further study in clinical setting.

## MATERIALS AND METHODS

### Cell lines, chemicals, and antibodies

GIST430 and GIST48 cells encoding the exon 11^V560_L576del (het)^/13^V654A^ and exon 11^V560D^/17^D820A^ mutant KIT oncoprotein, respectively, were gifts from Dr. Jonathan Fletcher (Harvard Medical School, USA). AUY922 was kindly supplied by Novartis. Rapamycin, 3-MA, bafilomycin A_1_, CelLytic^TM^ cell lysis reagent, and rabbit anti-MAP1LC3A/B were purchased from Sigma Aldrich. Primary antibodies against KIT and phospho-KIT (Tyr703) were obtained from DAKO and Invitrogen, respectively. Antibodies against RPS6KB1, phospho-RPS6KB1 (Thr389), MAPK1/3, phospho-MAPK1/3 (Tyr204/Thr202), AKT, phospho-AKT (Ser473), BECN1, and ATG5 were purchased from Cell Signaling Technology. Normal rabbit IgG, normal goat IgG, normal mouse IgG, goat anti-MAP1LC3B, rabbit anti-PARP1, and mouse anti-SQSTM1 were obtained from Santa Cruz Biotechnology. Antibodies against HSPA1A and ACTIN were obtained from Stressgen and Millipore, respectively.

### RNA interference

Small interfering RNA (siRNA) specific to human *BECN1* and negative control siRNA were purchased from Invitrogen. Specific siRNA targeted to human *ATG5* was synthesized by Invitrogen using the published sequence 5-GGACGAAUUCCAACUUGUU-3 [17]. GIST430 and GIST48 cells were transfected with siRNA of *BECN1*, *ATG5*, or negative control using Lipofectamine 2000® (Invitrogen) according to the manufacturer's protocol. Briefly, GIST430 and GIST48 cells at 90% confluence in 6-well plates were transfected with the indicated dose of annealed RNA duplexes mixed with 2 μL of Lipofectamine 2000^®^ for 6 h, and then replaced with serum-containing complete medium for 72 h. The influence of siRNA on BECN1 or ATG5 protein expression was confirmed by immunoblotting with specific antibodies.

### Cell viability assay

For this assay, 5×10^4^ GIST430 or 4×10^4^ GIST48 cells were seeded in each well of 24-well plates. GIST430 and GIST48 cells were exposed to various concentrations of drugs for 4 days. The methylene blue dye assay was used to evaluate the effects of each drug on the relative number of viable cells [17]. Data were measured with a SpectraMax M5 microplate reader (Molecular Device) at 595 nm and normalized to the DMSO-only control group. The IC_50_ was determined by plotting growth relative to that of the untreated controls. All experimental points were measured in duplicate wells for each plate and replicated in at least three plates.

To assess the interaction between AUY922 and rapamycin, the combination index (CI) was analyzed using the method of Chou and Talalay, based on the multiple drug effect equation [51]. Briefly, the constant ratio combination design was chosen to assess the combination effect of both drugs. Dose-response curves were determined with both drugs in combination at a fixed ratio equivalent to the ratio of their IC_50_ values. The CI value indicates that the effects of the two drugs were additive (CI>1), synergistic (CI<1), or antagonistic (CI>1). All experimental points were measured in duplicate wells for each plate and were replicated in at least three plates.

### Clonogenic assay

1×10^3^ GIST430 or GIST48 cells in the logarithmic growth phase were seeded in each well in 6-well plates. GIST430 and GIST48 cells were incubated with the indicated doses of drugs for 1 day and then replaced with growth media for an additional 14 days. The cells were stained with 50% ethanol containing 0.5% methylene blue for 5 h. The plates were washed five times with water and allowed to air-dry. Colonies were countered manually. The IC_50_ value corresponding to 50% inhibition of cell growth was determined after plotting growth relative to the untreated controls. Each value represented the average of at least three independent experiments performed in triplicate.

### Immunoblotting studies

Cells were lysed in CelLytic^TM^ M Cell Lysis Reagent containing protease inhibitors and phosphatase inhibitors. The protein concentration was determined using the Bradford method (Bio-Rad). Sodium dodecyl sulfate-polyacrylamide gel electrophoresis (SDS-PAGE) was performed after loading equal amounts of protein into each lane. Proteins were transferred to polyvinylidene fluoride (PVDF) membranes for immunoblotting. After blocking with bovine serum albumin (BSA), the membranes were blotted by adding primary antibodies against specific proteins, followed by the appropriate secondary antibodies. Protein bands were detected by enhanced chemiluminescence (PerkinElmer), developed by autoradiography and quantified using 1Dscan EX gel analysis software (Scanalytics). The data are expressed as the mean ± S.E.

### Immunofluorescence staining

For immunofluorescence staining, GIST430 and GIST48 cells were fixed with paraformaldehyde, permeabilized with Triton X-100, and blocked with 5% BSA in PBS at 4°C overnight. Cells were incubated with primary antibodies against KIT, MAP1LC3B, or SQSTM1. Cells used as nonspecific staining control were incubated with normal rabbit, goat, or mouse antibodies. The cells were washed three times with PBS, incubated with a fluorescently labeled secondary antibody, washed three times with PBS, and examined under a confocal microscope (FV1000, Confocal Laser Scanning Biological Microscope, Olympus).

### Flow cytometry

Apoptosis was determined by staining cells with Annexin V-FITC and PI (BD Biosciences) according to the manufacturer's protocol, followed by flow cytometry analysis. Briefly, GIST430 and GIST48 cells were incubated with drugs for the indicated times and doses and then trypsinized. Each sample containing 1×10^5^ cells was washed with cold PBS and resuspended in 100 μl binding buffer. Then, 5 μl Annexin V-FITC and 5 μl PI were added to the cells, which were then incubated for 15 min at room temperature (RT) in the dark. An additional 400 μl binding buffer was added to the reaction prior to analysis.

### GIST430 xenograft animal model and the drug treatment

NOD/SCID mice were purchased from LASCO. The animals were subcutaneously implanted with 2×10^7^ GIST430 cells mixed with equal volume of Matrigel (BD Biosciences) in 0.1 mL in one flank per mouse. Tumor volume was estimated from caliper measurements every two days after implantation and calculated as 1/2 x length (mm) x width^2^ (mm). After tumors developed for approximately 4 weeks and tumor volumes reached 50 to 100 mm^3^, mice were intraperitoneally injected with the indicated dose of AUY922 and rapamycin dissolved in DMSO, either alone or in combination, twice a week for 4 consecutive weeks. The control group was treated with DMSO only. Tumor volumes were measured every two days after drug treatment. At the end of the experiments, animals were sacrificed and tumors were visualized and stored at −80°C for further immunohistochemical analysis.

### Immunohistochemical analysis

Sections of tumor tissue blocks were cut onto adhesive-coated glass slides (Instrumedics, Hackensack) at a thickness of 3 μm. The slides were dewaxed in xylene and rehydrated through a graded alcohol series. Then, slides were pressure-cooked in 10 mM citrate buffer (pH 6) for 7 minutes for antigen retrieval and washed using TBS with 0.1% Tween 80 for 5 minutes. Endogenous peroxidase activity was blocked by 3% H_2_O_2_ treatment. After washing, the slides were incubated with primary antibodies against KIT (Epitomics), MAP1LC3B, HIF1A (Novus Biologicals), and PECAM1 (BD Biosciences) at RT for 1 hour. Primary antibodies were detected following the user's manual of the ChemMate DAKO EnVision kit (DAKO). The slides were incubated with the secondary antibody for 30 minutes and developed with 3,3-diaminobenzidine for 5 minutes. Slides were then counterstained with hematoxylin. Incubation without the primary antibody was used as a negative control.

### Statistical analysis

We analyzed the data with the Statistical Package for the Social Science (Software version 16 for Windows (SPSS, Inc.)). All data are expressed as the mean ± SEM. Comparison of means between the groups was performed by one-way analysis of variance (ANOVA) followed by the least significant difference (LSD) test. The level of significance was set at *p* < 0.05.
